# Limited Fitness Advantages of Crop-Weed Hybrid Progeny Containing Insect-Resistant Transgenes (*Bt/CpTI*) in Transgenic Rice Field

**DOI:** 10.1371/journal.pone.0041220

**Published:** 2012-07-17

**Authors:** Xiao Yang, Feng Wang, Jun Su, Bao-Rong Lu

**Affiliations:** 1 Ministry of Education Key Laboratory for Biodiversity Science and Ecological Engineering, Department of Ecology and Evolutionary Biology, Fudan University, Shanghai, China; 2 Fujian Province Key Laboratory of Genetic Engineering for Agriculture, Fujian Academy of Agricultural Sciences, Fuzhou, China; Ghent University, Belgium

## Abstract

**Background:**

The spread of insect-resistance transgenes from genetically engineered (GE) rice to its coexisting weedy rice (*O. sativa f. spontanea*) populations *via* gene flow creates a major concern for commercial GE rice cultivation. Transgene flow to weedy rice seems unavoidable. Therefore, characterization of potential fitness effect brought by the transgenes is essential to assess environmental consequences caused by crop-weed transgene flow.

**Methodology/Principal Findings:**

Field performance of fitness-related traits was assessed in advanced hybrid progeny of F_4_ generation derived from a cross between an insect-resistant transgenic (*Bt/CpTI*) rice line and a weedy strain. The performance of transgene-positive hybrid progeny was compared with the transgene-negative progeny and weedy parent in pure and mixed planting of transgenic and nontransgenic plants under environmental conditions with natural *vs.* low insect pressure. Results showed that under natural insect pressure the insect-resistant transgenes could effectively suppress target insects and bring significantly increased fitness to transgenic plants in pure planting, compared with nontransgenic plants (including weedy parent). In contrast, no significant differences in fitness were detected under low insect pressure. However, such increase in fitness was not detected in the mixed planting of transgenic and nontransgenic plants due to significantly reduced insect pressure.

**Conclusions/Significance:**

Insect-resistance transgenes may have limited fitness advantages to hybrid progeny resulted from crop-weed transgene flow owning to the significantly reduced ambient target insect pressure when an insect-resistant GE crop is grown. Given that the extensive cultivation of an insect-resistant GE crop will ultimately reduce the target insect pressure, the rapid spread of insect-resistance transgenes in weedy populations in commercial GE crop fields may be not likely to happen.

## Introduction

The commercial application of genetically engineered (GE) crops in agricultural production has aroused great biosafety concerns worldwide. The potential environmental impacts caused by the cultivation of the GE crops are the most debated issues [1]–[3]. Transgene flow from a GE crop into populations of wild or weedy relatives and its potential ecological risks is considered as a key environmental problem [4]–[6]. (Trans) gene flow from a crop to its wild relatives has been widely documented in the last decades [7]–[9]. However, our knowledge on the role of introgressed transgenes that confer novel traits with a strong selective advantage in changing the evolutionary process of wild or weedy populations is still limited. Therefore, assessing potential environmental risks caused by the extensive cultivation of GE crops prior to their commercialization becomes a common practice [1], [5], [8]. The study of potential ecological consequences created by transgene flow to wild relatives particularly the coexisting and conspecific weeds will provide solid bases for environmental risk assessment [10]–[13]. The fate of weedy populations that acquired transgenes through gene flow is largely different, depending on the fitness effect of the introgressed transgenes under given environmental conditions [5], [14]. If the introgressed transgenes can increase fitness, the transgenes will enhance the competitiveness and invasiveness of the weedy populations, leading to the rapid spread of the transgenes in the weedy populations, and *vice versa*. Thus, estimating fitness effect of transgenes on weedy populations is essential.

Rice is an important world crop providing staple food for nearly one half of the global population [15]. Research and development of GE rice has been extensively practiced in China, and consequently a many GE rice lines with novel traits such as insect-resistance, herbicide-tolerance, and improved grain quality have been developed [5], [11], [16]. In 2009, Chinese authority granted biosafety certificates for two insect-resistant rice lines containing a *Bt* transgene (*Bacillus thuringiensis*) [17], meaning that GE rice may enter commercial production in near future. Transgene spread from insect-resistant GE rice to its coexisting weedy rice populations through gene flow becomes a major environmental biosafety concern. Weedy rice (also known as red rice, *O. sativa f. spontanea*) is a noxious weed that causes significant losses of rice yield and quality worldwide [18], [19]. The introgression of transgenes with selective advantages may largely enhance the spread of the weedy rice, causing more serious weed problems. Weedy rice is an annual weed conspecific to cultivated rice [20], and transgene flow from cultivated rice to weedy rice cannot be avoided owing to their similar flowering phenology [21]. It is therefore valid to determine the fitness effect of insect-resistance transgenes on weedy rice populations under different insect pressure for risk assessment.

Fitness of an insect-resistance transgene is largely associated with the environment in which insect pressure can largely be variable [22]–[25]. The relationship between fitness effect brought by an insect-resistance transgene and the ambient insect pressure has not been well described. A number of studies of crop-weed hybrids showed variable fitness effect brought by insect-resistant transgenes under different insect occurrences [25], [26]. It is apparent based on these studies that the enhanced fitness benefit of insect-resistant transgenes was always associated with the high target herbivore pressure, whereas under low herbivore pressure the benefit brought by the insect-resistant transgenes was reduced. Therefore, to analyzed fitness effect brought by an insect-resistance transgene under designed experimental conditions with different insect pressure should be the key to detect the fitness effect of such a transgene.

Studies have already revealed the effects of *Bt* transgene in crop-wild or crop-weed hybrid progeny under different environments [23], [25], [26]–[28]. Our previous studies of F_1_–F_3_ crop-weed hybrids and their derived lineages from insect-resistant rice revealed increased fecundity brought by *Bt/CpTI* transgenes when target insects were abundant [25], [29]. However, the relationship between variation in different insect pressure and the corresponding fitness change was not well addressed by a properly designed experiment. In this study, we intended to address the following questions: (i) Are the insect-resistant transgenes (*Bt/CpTI*) still effective on resisting rice target herbivores in the transgenic F_4_ populations derived from a GE crop-weed hybrid under natural insect pressure? (ii) Does the transgenic F_4_ population have significantly higher fitness than the nontransgenic F_4_ population and weedy rice parent under natural insect pressure? (iii) Does the mixed planting of transgenic plants significantly reduce the ambient insect pressure of the nontransgenic plants, and consequently, reduce the potential fitness benefit brought by insect-resistance transgenes in the experimental fields? The answer of above questions is essential for assessing the long-term ecological impacts caused by insect-resistant transgene flow from to its weedy rice populations.

## Methods

### Experimental Materials and their Sources

Our experiment included two sets of F_4_ populations, with or without transgenes derived from a crop-weed hybrid, and its weedy rice parent. The F_4_ populations were generated by continued self-pollination from the F_1_ hybrids of an insect-resistant GE rice line crossed with a weedy rice strain (used as the pollen recipient) [25], [29]. The GE rice line produced by Fujian Academy of Agricultural Sciences in Fuzhou of China had the *Bt/CpTI* (*Bacillus thuringiensis* (*cryIAc*) and cowpea trypsin inhibitor) transgenes in a double insertion, tightly linked with the selectable marker gene *hyg* (hygromycin resistance), transformed by an agrobacterium method [30]. This GE rice line was produced to control lepidopteran pests such as rice stem borers (*Scirpophaga incertulas*, *Chilo suppressalis*, and *Sesamia inferens*) and rice leaf-folders (*Cnaphalocrocis medinalis*) [24]. The weedy rice strain (coded as W) was donated by Dr. H. S. Suh of Yeungnam University from S Korea [29]. The identification and separation of the transgene homozygous population (coded as TP) from non-transgene homozygous population (TN) were made from screening the seedlings derived from seeds of F_2_ plants using the hygromycin-B water solution treatment [25]. The selected transgene-positive and transgene-negative F_3_ populations were self-pollinated for one more generation. Therefore, plants of both transgene-positive and transgene-negative F_4_ populations were homozygous at the transgenic locus.

### Experimental Design

The field experiment was conducted in 2010 in the environment with a natural insect occurrence at the designated Biosafety Assessment Center in Fuzhou, Fujian Province, China. The experimental plots were allocated in two separate blocks (natural insect *vs*. low insect pressure) isolated from each other for >100 m. The block with natural insect pressure was free of any insecticide treatment, whereas the block with low insect pressure was achieved by spraying different insecticides (Methamidophos, Folimat, Buprofezin, and Monosultap) every 7–10 days starting at the tillering stage. The main comparison was made among the weedy parent, transgenic and nontransgenic hybrid lineages under the different insect pressure. Therefore, under each of the insect-pressure environmental conditions (natural *vs*. low), the following treatments were designated: (1) pure planting of weedy rice parent, transgene-positive, and transgene-negative F_4_ populations; (2) mixed planting between transgene-positive and -negative F_4_ populations, or transgene-positive F_4_ population and weedy rice parent, or transgenic-negative F_4_ population and weedy rice parent. Consequently, there were a total of twelve treatments included in the experiment.

For each treatment, four replicates (plots) were included. In the pure planting design, each plot included 64 plants in an 8×8 grid with 20 cm spacing between plants. In the mixed planting design, each plot included 32 plants of either type of the experimental materials grown alternatingly in an 8×8 grid with 15 cm spacing between plants. The field layout of plots was arranged in a complete randomized design in the two blocks. Seeds of all the plant materials were germinated in 50×100 cm pots filled with paddy soil from fields and placed in a green house. Seedlings were transferred to a nursery bed 20 days after seed germination, and then transplanted into field plots ∼40 days after seed germination. To avoid accidental seed shattering, panicles of all plants were enclosed by nylon mesh-bags 10–15 days after the flowering.

**Figure 1:**
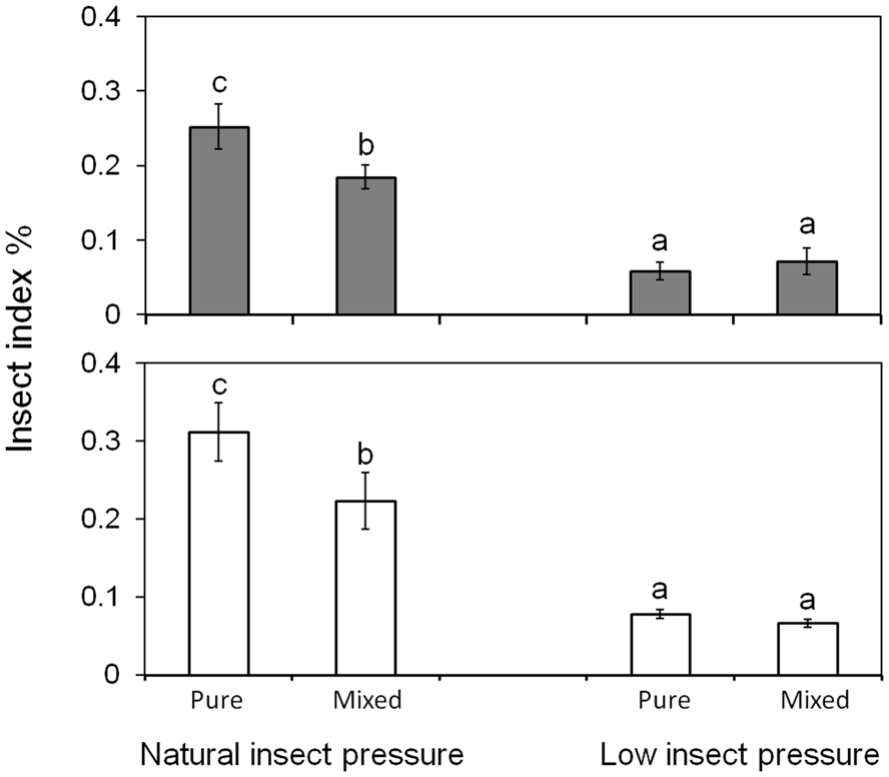
Insect index (%) calculated based on the ratio of blasted tillers and folded leaves on weedy parent (above) and transgene-negative hybrid progeny (below) in different cultivation modes (pure *vs.* mixed) under natural or low insect pressure. The comparison was made among pure and mixed planting under natural insect and low insect pressure. Different letters above the columns indicate significant differences according to Duncan’s multiple range tests after Bonferroni correction. “Pure” indicates pure planting, “Mixed” indicates mixed planting. Bars represent standard error. Levels of significance: P<0.05.

### Data Collection and Analysis

For estimating insect damage and fitness-related traits, all plants in a plot were characterized at various vegetative and reproductive stages, except for those in the border rows that were excluded to avoid the edge effect. As a result, a total 36 plants from a pure-planting plot and 18 plants for each of the two types of populations from a mixed-planting plot were characterized. Blasted tillers (by rice stem borers) and folded leaves (by leaf-folder) at the beginning of flowering stage were measured to estimate the insect damage. An insect index was used to estimate the insect pressure that was calculated as the sum of percentage of both blasted tiller and folded leaves in the plots of weedy parent and nontransgenic F_4_ populations. The following fitness-related traits were included for measurement: plant height, number of tillers, number of panicles, and number of filled seeds per plant, seed set, and 1000-seed weight at various growth stages.

One way ANOVA (Duncan’s multiple range test) was used to examine significant differences for insect index obtained from weedy rice and transgene-negative plants in pure and mixed planting under natural and low insect pressure, and to test significant differences in insect damage and fitness-related traits among weedy parent, transgene-positive, and transgene-negative populations in pure planting, followed by the Bonferroni correction. The paired *t*-test was used to examine significant differences between transgene-positive and transgene-negative, transgene-positive and weedy parental, or transgene-negative and weedy parental populations in mixed planting. All statistical analyses were performed using the software package IBM SPSS Statistics ver. 19.0 for Windows (SPSS Inc., IBM Company Chicago, IL, USA, 2010).

## Results

### Insect Pressure Under the Two Environmental Conditions

The insect index calculated from the insect damage of blasted tillers and folded-leaves demonstrated a significant difference between the two blocks with natural insect pressure and low insect pressure (Fig. 1; Table 1 and 2). As estimated from the weedy parental and transgene-negative F_4_ populations in pure planting, the insect index was significantly higher under the natural insect pressure (25.3% and 31.5%) than that the under low insect pressure (5.8% and 7.8%; P<0.001). The significant differences of insect pressure allowed us to determine the net benefit caused by the transgenes when all the plants were exposed to herbivores, and the net cost caused by the transgenes when herbivores were absent or reduced to a low level.

**Table 1:** Average values and standard errors (±) of insect damage and fitness-related traits in weedy rice parent, transgene-positive, and transgene-negative crop-weed hybrid progeny (F_4_) in pure planting under natural (above) vs. low (below) insect pressure.

Trait	Plant material		
	Weedy parent	Transgene-positive	Transgene-negative
**Natural insect**			
Blasted tillers %	13.4±1.1 b	7.0±1.3 a	15.6±2.0 b
Folded leaves %	11.8±2.1b	1.5±0.3 a	15.6±3.1 b
Plant height (cm)	91.9±4.0 a	111.9±4.2 b	112.3±2.6 b
No. of tillers	17.3±1.1 a	18.2±0.3 a	16.0±1.2 a
No. of panicles	10.4±1.0 a	15.0±0.5 b	12.0±0.9 a
Seed set (%)	50.0±1.5 b	47.8±2.5 b	40.8±0.9 a
1000-seed weight (g)	20.3±0.2 a	21.4±0.3 b	20.7±0.2 ab
**Low insect**			
Blasted tillers %	4.8±1.2 a	5.0±0.8 a	6.5±0.7 a
Folded leaves %	1.0±0.0 b	0.2±0.2 a	1.3±0.2 b
Plant height (cm)	107.3±1.7 a	111.0±2.5 a	120.5±2.7 b
No. of tillers	13.9±0.6 a	13.3±0.4 a	13.0±0.9 a
No. of panicles	13.6±0.7 b	10.6±0.7 a	11.0±0.4 a
Seed set (%)	51.3±1.3 ab	52.2±1.4 b	48.0±0.8 a
1000-seed weight (g)	19.7±0.2 a	22.1±0.2 b	21.6±0.3 b

Different letters following the average values in the same rows indicate significant differences according to Duncan’s multiple range tests after Bonferroni correction (N = 4 plots).

**Table 2:** Average values and standard errors (±) of insect damage and fitness-related traits in weedy rice parent (W), transgene-positive (TP), and transgene-negative (TN) crop-weed hybrid progeny (F_4_) in mixed planting under natural (above) vs. low (below) insect pressure.

Trait	Plant material					
	TP *vs.* TN		TP *vs.* W		TN *vs.* W	
**Natural insect**						
Blasted tillers %	11.4±2.0	17.3±0.9*	9.6±2.1	13.0±2.3	12.9±1.4	18.1±3.0
Folded leaves %	2.9±1.7	5.0±3.0	4.2±1.3	5.4±1.2	13.6±2.7	10.3±3.7
Plant height (cm)	101.4±2.6	103.4±4.8	96.9±2.9	93.1±3.0	107.9±6.4	99.4±6.7
No. of tillers	11.0±1.0	9.9±0.8	13.2±1.2	12.0±1.0	11.8±0.5	9.8±1.0
No. of panicles	8.9±0.4	8.6±0.8	9.7±1.0	8.3±0.4	8.9±0.7	8.2±0.9
Seed set (%)	42.5±4.2	37.1±0.6	43.5±1.7	49.3±2.6	37.0±2.0	47.8±3.8*
1000-seed weight (g)	20.4±0.6	20.5±0.2	20.6±0.1	19.6±0.3*	21.1±0.6	19.1±0.3*
**Low insect**						
Blasted tillers %	5.4±0.3	5.8±0.4	5.3±1.5	6.5±1.9	5.7±2.2	8.2±1.7
Folded leaves %	0.4±0.3	0.8±0.3	0.4±0.1	0.7±0.2	2.3±0.4	0.7±0.3*
Plant height (cm)	112.3±5.0	116.7±3.4	104.5±4.7	98.5±3.4	111.4±2.8	104.1±1.4
No. of tillers	7.6±0.1	8.2±0.3	8.5±0.5	7.8±0.5	8.8±0.4	8.3±0.5
No. of panicles	6.6±0.4	7.2±0.5	6.8±0.4	6.2±0.3*	7.3±0.6	7.2±0.3
Seed set (%)	53.9±0.9	49.0±3.2	49.9±5.7	61.9±2.4	51.6±2.6	55.7±2.9
1000-seed weight (g)	22.3±0.8	21.9±0.5	21.9±0.7	19.3±0.4*	21.7±0.2	18.8±0.4**

Level of significance was calculated based on the paired *t*-test between TP and TN, TP and W, or TN and W (N = 4 plots).

* P<0.05,

** P<0.01.

Noticeably, the insect index was significantly lower in the mixed planting of transgene-positive populations than in the pure planting under natural insect pressure (Fig. 1), indicating that the mixed planting with transgene-positive plants will generally reduce the ambient insect pressure. However, such reduction of insect damage was not observed in the mixed planting of transgene-positive plants under low insect pressure.

### Performance of F_4_ and Weedy Parental Populations in Pure Planting

**Figure 2:**
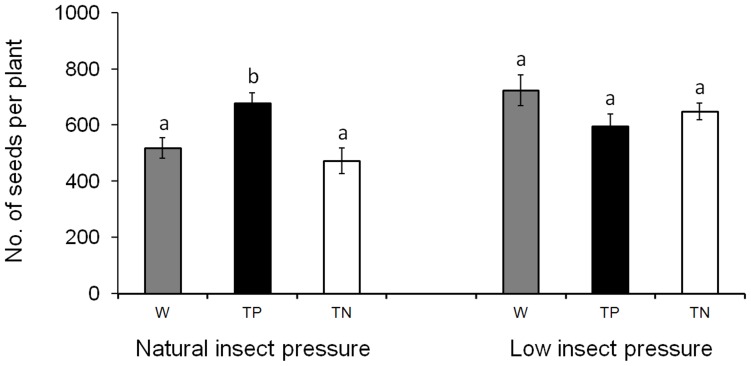
The average number of filled seeds per plant produced by weedy rice parent (W), transgene-positive (TP), and transgene-negative (TN) hybrid progeny under natural vs. low insect pressure in pure cultivation. The comparison was made among weedy rice parent, transgene-positive, and transgene-negative hybrid progeny. Different letters above the columns indicate significant differences according to Duncan’s multiple range tests after Bonferroni correction. Bars represent standard error. Levels of significance: P<0.05.

In general, transgene-positive F_4_ populations with insect-resistance transgenes showed significantly less insect damage than transgene-negative F_4_ populations and weedy parents, under natural insect pressure (Table 1). Transgene-positive F_4_ populations had a better performance for most of the fitness-related traits than transgene-negative F_4_ populations and weedy parents, under natural insect pressure (Table 1). For example, transgene-positive plants produced a significantly higher number of panicles (25% increase) and ratio of seed set (17% increase) than their transgene-negative plants (Table 1). Particularly, the number of seeds produced by transgene-positive plants was significantly higher than both transgene-negative plants (44% increase) and weedy parents (31% increase) (Fig. 2). Under low insect pressure, nearly all the fitness-related traits did not show significant differences between transgene-positive and transgene-negative populations (Table 1; Fig. 2) in both pure and mixed planting, suggesting no obvious fitness cost from the transgenes.

Noticeably, transgene-negative F_4_ populations showed significantly higher values of plant height and 1000-seed weight than their weedy rice parental populations under both natural and low insect pressures (Table 1). This indicated that crop-weed hybridization may bring benefit through the enhanced performance of some traits of hybrids and hybrid lineages in later generations.

### Performance of F_4_ and Weedy Parental Populations in Mixed Planting

In contrast to the pure planting, transgene-positive and transgene-positive F_4_ populations showed apparently less pronounced differences in insect damage in mixed planting than in pure planting under natural insect pressure (Table 2). The same trend was observed between transgene-positive F_4_ and weedy parental populations (Table 2) in mixed planting. As a result, transgene-positive F_4_ populations did not show significant differences for nearly all the fitness-related traits, compared with transgene-negative F_4_ and weedy parental populations under natural insect pressure (Table 2). These results indicated that the mixed-planting plots with transgene-positive plants would result in a reduced insect damage to transgene-negative and weedy rice plants, and consequently reduce the differences in fitness-related traits between transgene-positive and transgene-negative plants.

**Figure 3:**
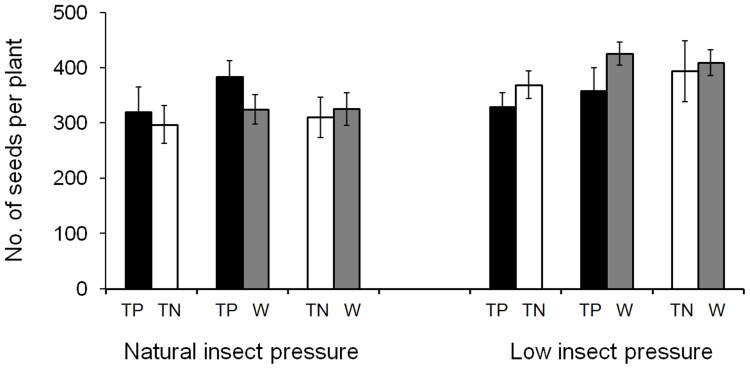
The average number of filled seeds per plant produced by weedy rice parent, transgene-positive (W), and transgene-negative (TP) hybrid progeny under natural vs. **low insect pressure in mixed cultivation.** The comparison was made between transgene-positive and transgene-negative, transgene-positive and weedy rice parent, or transgene-negative and weedy rice parent, based on the paired *t*-tests. Bars represent standard error. Levels of significance: P<0.05.

Similarly, under low insect pressure, nearly all the fitness-related traits did not show significant differences among transgene-positive F_4_, transgene-negative F_4_ and weedy parental populations (Table 2; Fig. 3). However, it is worthy of notice that the 1000-seed weight of transgene-positive and transgene-negative F_4_ plants was significantly higher than that of weedy parental plants, suggesting again the benefit of hybridization brought to hybrid progeny compared with their weedy parents in terms of seed weight, regardless of the presence of transgenes.

## Discussion

The key point of assessing potential ecological risks caused by an insect-resistant transgene introgressed to weedy populations is to analyze the increased fitness of transgenic plants in comparison with their nontransgenic counterparts derived from the same crop-weed hybridization under controlled environmental conditions [11], [22], [24], [25], [31]. Our data from this study indicated significant differences in insect pressure (as measured by the insect index, Fig. 1) between the two rice planting environments with natural *vs*. low insect pressure, achieved by artificial spray of insecticides. The insect pressure in both pure and mixed planting plots of weedy rice parents and transgene-negative F_4_ populations was significantly higher under the natural-insect condition than that under the controlled low-insect condition. These results indicate the effectiveness of insect control in our experiment. In addition, as indicated by the insect damage on weedy parents and transgene-negative plants, significantly higher insect index was also detected in pure planting plots than in the mixed-planting plots of nontransgenic plants (including parents) with transgenic plants under natural insect. In contrast, the mixed-planting plots with transgenic plants did not show such significant differences in insect index under low insect pressure. All together, these results demonstrate that the differential environment allowed us to analyze the fitness effect of insect-resistance transgenes in weedy rice plants against their nontransgenic counterparts, and that the mixed planting of transgenic plants in a field plot can significantly reduce the ambient insect pressure.

In pure planting, transgene-positive crop-weed F_4_ plants showed significantly greater values of fecundity-related traits, such as the number of panicles and well-filled seeds and the ratio of seed set than their transgene-negative plants under natural insect pressure. Particularly, the number of panicles and filled seeds of transgene-positive F_4_ plants showed significantly higher values than the weedy rice parents. However, such significant differences between the transgenic plants and nontransgenic plants were not detected in these traits under the low insect pressure. The increased fecundity of transgenic crop-weed F_4_ plants is most likely brought by the introgression of insect-resistance transgenes that significantly reduced insect attack to the plants. The increased fecundity in the transgenic F_4_ plants demonstrates that the introgressed insect-resistant transgenes (*Bt/CpTI*) are still effective on controlling rice target herbivores in the advanced generation of crop-weed hybrid populations. This result is consistent with our previous study in which the F_2_–F_3_ lineages derived from the crop-weed hybrids with the same transgenic event showed effective control of insect damages to the hybrid lineage compared with both transgene-negative hybrid lineages and the weedy parents, under a high level of insect pressure [25], [29]. Many previous studies on crop-weed and crop-wild hybrid lineages also demonstrated that insect-resistance transgenes can dramatically reduce the target herbivores and increase the fecundity in hybrids and their advanced generation of hybrid populations [23], [25], [29]. These findings are consistent with our results in this study. It seems possible to make a conclusion from the field experimental data of above studies including ours that the introgressed insect-resistance transgenes will maintain their strong ability to control herbivores and increase fecundity of transgenic populations derived from crop-weed or crop-wild transgene introgression. The transgenic plants may have a strong ability to compete with their nontransgenic counterparts including nontransgenic hybrid and parental populations [23], [25], [28].

However, it is necessary to point out that in the mixed-planting plots the fecundity traits (particularly seed production) of the transgene-positive plants did not show significantly increased values, compared to the transgene-negative plants and weedy rice parents under natural insect pressure. This result suggests apparent losses of fitness benefit that should have been brought by the insect-resistance transgenes in mixed planting under natural insect pressure. The similar phenomenon was also observed in a number of studies in which the fitness benefit and cost of insect-resistant GE rice lines and their nonGE parental lines was analyzed under natural and low insect pressure, respectively [22], [24]. Associated with the data of insect index from this study where significantly lower insect pressure was recorded in the mixed planting plots than in the pure planting plots under the same environmental condition of natural insect (Fig. 1), we think that the loss of fitness benefit to insect-resistant transgenic crop-weed hybrid populations in mixed planting is most likely owing to the considerably reduced insect pressure. In other words, the generally low ambient insect pressure in the experimental field caused by the mixture of insect-resistant transgenic plants will significantly reduce the potential fitness advantages that should have been brought by insect-resistance transgenes. As a result, the expected long-term persistence and rapid spread of insect-resistance transgenes in weedy rice populations caused considerably increased fitness advantages following transgene flow from a GE rice variety may not happen in the realistic situation if such fitness advantages are extremely limited. Under the actual situation where an insect-resistant GE rice variety is cultivated, the ambient insect pressure in an extensive field area should be reduced to a much greater extent than that in our experimental plots. The spread of transgenes in weedy rice populations might be considerably limited owning to the negligible fitness increase in insect-resistance GE rice fields where the target insect pressure is significantly reduced, although the issue of transgene flow from an insect-resistant GE rice variety to its coexisting weedy rice populations should not be neglected.

Our finding has its important implications for the risk assessment of transgene flow from insect-resistant GE crop to its wild relatives, and to the conspecific weedy populations in particular. Given a determined frequency of transgene flow, the magnitude of potential environmental risks should largely depend on the fitness effect of a transgene. It is generally recognized that fitness effect of an insect-resistance transgene is determined by the ambient insect pressure in the environment where wild/weedy populations occur. Therefore, the assessment of environmental risks caused by transgene flow from an insect-resistant GE crop to wild populations should first consider the pressure of target insects in the concerned environment. This principle may also applied to the risk assessment of transgene flow to wild populations, considering that insect pressure in natural habitats is significantly lower than in agriculture habitats [32]. For the crop conspecific weedy populations co-occurring with a crop (and GE crop), crop-to-weed transgene flow cannot be avoided. The expected environmental impact from transgene flow from insect-resistant GE crop could be large because hypothetically insect-resistance transgenes will bring fitness benefit to the weedy populations. However, the fitness advantages might be limited due to the fact that weedy plants will be surrounded by insect-resistant plants in a GE crop field, reducing the ambient pressure of target herbivores significantly. Consequently, the spread of the transgenes in weedy populations would be limited. In reality, the extensive commercial cultivation of an insect-resistant GE crop will largely reduce target herbivores in a GE deployed area [33]. Under such a circumstance, the environmental impacts caused by the crop-to-weed transgene flow from an insect-resistant GE crop will also be limited.
